# Sepsis-induced Hyperleukocytosis in a Preterm

**DOI:** 10.7759/cureus.5594

**Published:** 2019-09-08

**Authors:** Emad U Alatassi, Marah Sukkar, Fadi N Garrada

**Affiliations:** 1 Vascular Surgery, Al Noor Specialist Hospital, Makkah, SAU; 2 Internal Medicine, Al-Moosa Specialist Hospital, Alehsaa, SAU; 3 General & Laparoscopic Surgery, Al Noor Specialist Hospital, Makkah, SAU

**Keywords:** leukocytosis, hyperleukocytosis, leukemoid reaction, sepsis, leukostasis, neonate, preterm

## Abstract

Hyperleukocytosis is defined as a white blood cell (WBC) count of ≥ 100,000/µL. Leukostasis refers to symptomatic hyperleukocytosis and is considered a medical emergency. In pediatric practice, hyperleukocytosis is most commonly described in leukemia and other myeloproliferative disorder, but other etiologies, such as infection, are less commonly mentioned.

In this case report, a one-day-old, preterm, male baby (26 weeks of gestation) was referred for preterm care. A sepsis-induced leukemoid reaction hyperleukocytosis diagnosis was presumed, and he was successfully treated with an empirical antibiotic with a gradual improvement in WBC counts.

## Introduction

Leukocytosis is commonly seen as a physiological or infectious response in neonates, but the white blood cell (WBC) count rarely exceeds 30,000/µL. Hyperleukocytosis is when the WBC count is over 100,000/µL, with one-week mortality that could reach 40% especially when WBCs exceed 300,000/µL [1]. Hyperviscosity syndrome can manifest as leukostasis by causing thrombosis, bleeding, or disseminated intravascular coagulation (DIC), particularly affecting intracerebral and pulmonary circulations [2].

By identifying and treating the primary cause, lowering the WBC count, preventing hyperviscosity and tumor lysis syndrome, all under close monitoring, we can possibly prevent or reduce complications.

We report here a case of sepsis-induced hyperleukocytosis in a preterm neonate and discuss the differential diagnoses with a brief literature review.

## Case presentation

A preterm male (26 weeks of gestation), born to a 31-year-old middle eastern multigravida mother by normal vaginal delivery, was referred for preterm care on Day 1. The mother had poor antenatal care and had not received antenatal steroids. There was no ABO or Rh incompatibility. On examination, the baby weighed 900 gm and had no obvious dysmorphic feature. He was lethargic, tachypneic, with intercostal retractions.

Investigations revealed Hb 10.5 g/dl and WBC count 159,000/µL(64% neutrophils). His platelet count was 258,000/µL while C-reactive protein (CRP) was negative. The complete metabolic panel, coagulation studies, and chest X-ray were within normal limits.

The baby was started on supportive care, fluid, and IV antibiotics (ampicillin+gentamycin). WBC counts were repeated on Day 4 and showed an increasing WBC count (185,500/ µL) with predominant neutrophils. Blood and cerebrospinal fluid (CSF) cultures were taken, and antibiotics were changed empirically to vancomycin and meropenem. The result of blood and CSF cultures came back negative. Peripheral blood smear and bone marrow aspiration were requested; the results were not suggestive of leukemia. Sepsis was presumed clinically to be the promoter of hyperleukocytosis. No source of infection could be identified.

The patient was closely monitored for intracranial hemorrhage, respiratory failure, hyperuricemia, renal failure, and other known complications of hyperleukocytosis. The WBC counts were closely monitored and repeated every other day; they showed a dramatic reduction within five days of starting vancomycin and meropenem and were eventually 14,700/µL on the 15th day of life. The patient responded well to supportive care and was discharged after 62 days of hospital stay.

## Discussion

The normal leukocyte count in neonates is physiologically higher than in adults with a range from 9000-30,000/µL [3]. Leukemoid reactions are known to be caused by infections, malignancies, hemolysis, hemorrhage, medications, and others.

Although a few conditions can present with elevated WBCs at such an early age, the major causes are congenital leukemia and leukocyte adhesion disorder. Another known cause of leukocytosis is a transient myeloproliferative disorder, which is reported in about 10% of Down syndrome cases [4-6].

Hyperleukocytosis caused by a severe leukemoid reaction can be presumed when leukemia has been ruled out. The exact etiology and mechanism are unknown, and a differential diagnosis is usually challenging. However, this can occur as a result of infections (Streptococcus agalactiae, Escherichia coli, Listeria monocytogenes, Clostridioides difficile, and others), carcinomas, severe hemorrhage, following exposure to certain drugs, such as corticosteroids, and it also has been reported in preterm infants without any identifiable cause [7-9]. A negative CRP does not rule out the possibility of bacterial infection in children [10].

The diagnostic workup consists of the exclusion of leukemias and the detection of an underlying cause. It should include a complete blood count, peripheral blood smear, bone marrow biopsy, blood culture, CSF fluid analysis and culture, complete metabolic panel, coagulation studies, and chest X-ray.

Hyperleukocytosis might cause severe, life-threatening complications, including leukostasis, thrombosis, DIC, intracranial hemorrhage, pulmonary hypertension, intrapulmonary hemorrhage, heart failure and hypoxemia, tumor lysis syndrome, and acute renal failure [11-12]. The main goal of hyperleukocytosis management is cytoreduction and decreasing blood viscosity to prevent complications. Management includes close monitoring with aggressive hydration, maintaining good diuresis, prevention of tumor lysis syndrome and DIC, and correction of any metabolic abnormalities.

In literature differentiating hyperleukocytosis from leukemia, cytoreduction can be achieved by either leukapheresis or exchange transfusion. Leukapheresis is the treatment of choice in symptomatic hyperleukocytosis but poses a higher risk and complications. An exchange transfusion is often a more practical option, easier and much safer than leukapheresis, especially when hyperleukocytosis is complicated by severe anemia [13-14]. Leukapheresis often is recommended for hyperleukocytosis because of its quick cytoreductive effect [15]. However, a recent study of an adult cohort failed to demonstrate that leukapheresis is associated with an improved early mortality rate and a similar study on the pediatric population concluded the same [16-18].

In our case, hyperleukocytosis was probably caused by sepsis. This is supported by the dramatic response to the antibiotic, despite having no pathogen isolated, as a negative culture does not exclude sepsis in neonates [19].

## Conclusions

The hyperleukocytosis and leukostasis mechanisms are poorly understood, and making a diagnosis is challenging. A negative culture and a negative CRP does not exclude infection or sepsis in a neonate. Management starts with close monitoring, followed by supportive care and directions to identify and treat the underlying cause and to prevent complications by good hydration and a proper empirical antibiotic selected based on the suspected pathogen. Leukapheresis should only be considered in symptomatic patients.

## References

[1] Porcu P, Cripe LD, Ng EW, Bhatia S, Danielson CM, Orazi A, McCarthy LJ (2000). Hyperleukocytic leukemias and leukostasis: a review of pathophysiology, clinical presentation and management. Leuk Lymphoma.

[2] Lester TJ, Johnson JW, Cuttner J (1985). Pulmonary leukostasis as the single worst prognostic factor in patients with acute myelocytic leukemia and hyperleukocytosis. Am J Med.

[3] Kliegman R, Behrman R, Jenson H, Stanton B (2007). Reference ranges for laboratory tests and procedures. Nelson Textbook of Pediatrics.

[4] Alizadeh P, Rahbarimanesh AA, Bahram MG, Salmasian H (2007). Leukocyte adhesion deficiency type 1 presenting as leukemoid reaction. Indian J Pediatr.

[5] Zwaan MC, Reinhardt D, Hitzler J, Vyas P (2008). Acute leukemias in children with down syndrome. Pediatric Clin North Am.

[6] Ishii E, Oda M, Kinugawa N (2006). Features and outcome of neonatal leukemia in Japan: experience of the Japan Infant Leukemia Study Group. Pediatr Blood Cancer.

[7] Wirbelauer J, Thomas W, Siauw C, Wössner R, Speer CP (2008). Leukemoid reaction in extremely immature preterm infants [Article in German]. Z Geburtshilfe Neonatol.

[8] Sakka V, Tsiodras S, Giamarellos-Bourboulis E, Giamarellou H (2006). An update on the etiology and diagnostic evaluation of a leukemoid reaction. Eur J Intern Med.

[9] Ganti AK, Potti A, Mehdi S (2003). Uncommon syndromes and treatment manifestations of malignancy. Case 2. Metastatic non-small-cell lung cancer presenting with leukocytosis. J Clin Oncol.

[10] Kono T, Otsuka M, Ito M (2019). Negative C‐reactive protein in children with bacterial infection. Pediatr Int.

[11] Simonsen KA, Anderson-Berry AL, Delair SF, Davies HD (2014). Early-onset neonatal sepsis. Clin Microbiol Rev.

[12] Ruggiero A, Attinà G, Piastra M, Maurizi P, MastrangeloS MastrangeloS, Pietrini D, Riccardi R (2009). Severe hyperleukocytosis and multifocal intracranial haemorrhage: not always a fatal outcome. Int J Hematol.

[13] Paddock CD, Sanden GN, Cherry JD (2008). Pathology and pathogenesis of fatal Bordetella pertussis infection in infants. Clin Infect Dis.

[14] Weng YH, Chiu YW (2011). Comparison of efficacy and safety of exchange transfusion through different catheterizations: femoral vein versus umbilical vein versus umbilical artery/vein. Pediatr Crit Care Med.

[15] Jain R, Bansal D, Marwaha RK (2013). Hyperleukocytosis: emergency management. Indian J Pediatr.

[16] Bunin NJ, Kunkel K, Callihan TR (1987). Cytoreductive procedures in the early management in cases of leukemia and hyperleukocytosis in children. Med Pediatr Oncol.

[17] Porcu P, Danielson CF, Orazi A, Heerema NA, Gabig TG, McCarthy LJ (1997). Therapeutic leukapheresis in hyperleucocytic leukaemias: lack of correlation between degree of cytoreduction and early mortality rate. Br J Haematol.

[18] Sung L, Aplenc R, Alonzo TA, Gerbing RB, Gamis AS (2012). Predictors and short-term outcomes of hyperleukocytosis in children with acute myeloid leukemia: a report from the Children's Oncology Group. Haematologica.

[19] Korkmaz S (2018). The management of hyperleukocytosis in 2017: do we still need leukapheresis?. Transfus Apher Sci.

